# Empowering health care innovations: A look forward into 2024

**DOI:** 10.1002/hcs2.85

**Published:** 2024-02-22

**Authors:** Zongjiu Zhang, Tien Yin Wong, Haibo Wang, Jiefu Huang

**Affiliations:** ^1^ Institute for Hospital Management Tsinghua University Beijing China; ^2^ Tsinghua Medicine Tsinghua University Beijing China; ^3^ The First Affiliated Hospital of Sun Yat‐Sen University Guangzhou China; ^4^ China National Organ Donation and Transplantation Committee Beijing China

As we unveil the first issue of *Health Care Science* in 2024, it's a moment of reflection and anticipation. Under the astute guidance of our leadership and the collective wisdom of our distinguished Editorial Board Members, we've journeyed through an extraordinary year, marking significant milestones and embracing challenges with resilience and innovation. We believe our progress reflects not just a testament to our commitment but also an indication of our role in driving healthcare innovation and excellence.

**International diversity:** 2023, our journal emerged as a vibrant international academic platform, attracting manuscripts from 29 countries and regions. Focusing on technological innovation in healthcare services, we published six issues of 39 articles in 2023, reaching over 41,000 downloads from 166 countries and regions. This diversity is a clear indicator of our expanding global footprint and the trust the academic community places in us. The growth in our publications, alongside a dramatic increase in international manuscript submissions, highlights our journal's rising stature on the global healthcare community.

**Impactful publications:** 2023 saw us publishing groundbreaking work in the fields of healthcare management, healthcare policy, medical technology, and public health. We also delved into hot topics like xenotransplantation, gene editing, and the application of large language models in healthcare. Moreover, we published influential guides and research on technological responses to pandemics. These contributions include some of our most downloaded and cited articles since the launch of *Health Care Science*.

**Milestone recognitions:** The landmark achievements in 2023 were our inclusion in prestigious databases such as Scopus and the Directory of Open Access Journals (DOAJ), a recognition that came just a year after our launch. These inclusions are not just an honor but a responsibility to uphold the standards of scientific excellence and accessibility.

**Key metrics of publication process:** We are pleased to share some key metrics regarding our manuscript process. Our median time to first decision was 24 days and the median time to final decision stood at 37 days, demonstrating our commitment to providing timely feedback to authors while also reflecting our thorough review process. Overall, our acceptance rate in 2023 was 54%. For those articles accepted, the median time from acceptance to online publication was 46 days. We are actively optimizing our publication cycle to enhance efficiency, while firmly safeguarding the rigor of the peer review process. Our emerging Associate Editor (eAE) team, composed of 21 dynamic young talents in academia, has been instrumental in manuscript sourcing, reviewing, curated readings, and promotions, significantly contributing to our journal's success.

**Goals for 2024:** Looking ahead, we aim to streamline our editorial process while continuing to provide authors with comprehensive feedback. Engaging more closely with our editorial board and emerging editorial board members, both online and in person, will be a priority. Additionally, the introduction of a reviewer recognition program will celebrate the invaluable contributions of our academic gatekeepers, further enhancing the quality and efficiency of our publication process.

**Ongoing commitment:** Since the inception of *Health Care Science*, we remain committed to not just being another medical journal but a platform for holistic advancement in healthcare. Our dedication to advancing technology innovation, ensuring healthcare quality, prioritizing patient safety, and enhancing health system efficiency will remain the driving force behind our efforts. We firmly believe that the synergy of technology and healthcare will truly advance the science in healthcare.

Welcome to a year of possibilities, innovation, and dynamic strides in healthcare excellence!


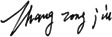


Editor‐in‐Chief

Zongjiu Zhang, MD


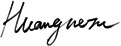


Honorary Editor‐in‐Chief

Jiefu Huang, MD


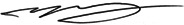


Honorary Editor‐in‐Chief

Wong Tien Yin, MD, PhD


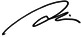


Executive Editor‐in‐Chief

Haibo Wang, MBBS, MSc, MPH

## AUTHOR CONTRIBUTIONS

**Zongjiu Zhang**: Conceptualization (equal); writing original draft (equal); review (equal). **Tien Yin Wong**: Conceptualization (equal); review (equal); editing (supporting). **Haibo Wang**: Conceptualization (supporting); writing original draft (equal); review (equal); editing (lead). **Jiefu Huang**: Review (equal); editing (supporting).

## CONFLICT OF INTEREST STATEMENT

The authors declare no conflict of interest.

## ETHICS STATEMENT

None.

## INFORMED CONSENT

None.

## Data Availability

Data sharing not applicable to this article as no datasets were generated or analyzed during the current study.

